# Effect of Black Tea Powder on Antioxidant Activity and Gel Characteristics of Silver Carp Fish Balls

**DOI:** 10.3390/gels9030215

**Published:** 2023-03-11

**Authors:** Jinling Hong, Jiaying Wu, Yanhong Chen, Zedong Jiang, Yanbing Zhu, Zhipeng Li, Xianmu Chen, Hui Ni, Mingjing Zheng

**Affiliations:** 1 College of Ocean Food and Biological Engineering, Jimei University, Xiamen 361021, China; 2 Collaborative Innovation Center of Seafood Deep Processing, Dalian Polytechnic University, Dalian 116034, China; 3 Fujian Provincial Key Laboratory of Food Microbiology and Enzyme Engineering, Xiamen 361021, China; 4 Research Center of Food Biotechnology of Xiamen City, Xiamen 361021, China; 5 Dongshan Tengxin Food Co., Ltd., Zhangzhou 363400, China

**Keywords:** black tea powder, fish balls, antioxidant activity, gel characteristics

## Abstract

The effect of black tea powder on the antioxidant activity and gel characteristics of fish balls from silver carp were investigated after freezing storage for 7 days. The results show that black tea powder with different concentrations of 0.1%, 0.2% and 0.3% (*w*/*w*) could significantly increase the antioxidant activity of fish balls (*p* < 0.05). In particular, at the concentration of 0.3%, the antioxidant activity was the strongest among these samples, where the reducing power, DPPH, ABTS and OH free radical scavenging rate were up to 0.33, 57.93%, 89.24% and 50.64%, respectively. In addition, black tea powder at the level of 0.3% significantly increased the gel strength, hardness and chewiness while greatly reducing the whiteness of the fish balls (*p* < 0.05). ESEM observation found that the addition of black tea powder could promote the crosslinking of proteins and reduced the pore size of the gel network structure of the fish balls. The results suggest that black tea powder could be used as a natural antioxidant and gel texture enhancer in fish balls, which we found to be much related to the phenolic compounds of black tea powder.

## 1. Introduction

Silver carp (*Hypophthalmichthys molitrix*), a widely cultivated and processed freshwater fish, is often used as a raw material for frozen surimi and surimi products [1]. Surimi products are gel products of fish myofibril protein with a wide variety of types, e.g., fish balls. Fish balls are the most representative surimi products, which are popular among consumers because of their high protein, low fat, and convenient eating properties. However, surimi products are prone to accumulate oxygen free radicals due to changes in physical and chemical properties during processing and freezing storage, triggering the oxidation of proteins and lipids. Excessive protein oxidation tends to hinder protein unfolding and reducing, altering the functional properties (e.g., gelation, hydrophobicity, and solubility) of the protein, resulting in a decline in quality and nutrition of surimi products, reducing the acceptability to the consumer [2,3]. In order to effectively control the protein oxidative denaturation of surimi products, some exogenous antioxidants such as fucoidan [4] and green tea extract [5] have been used in surimi products to improve the antioxidant activity and maintain the gel quality of surimi products. Currently, with the rise of health awareness, consumers are paying more attention to the use of naturally occurring antioxidant substances in their food [6]. However, such substances contain complex components such as proteins, polysaccharides and polyphenols, and how they affect the function and quality of surimi products still needs study.

Black tea [*Camellia sinensis* (L.) O. Kuntze (family: *Theaceae*)] (tea polyphenol content of 22.40%) is one of the most widely consumed teas in China, accounting for 78% of the world’s tea consumption [7]. It is beneficial to human health, with cholesterol-lowering, anti-cancer, anti-obesity, and other effects [8]. The bioactive substances of most concern in black tea are oxidized phenolic compounds, including thearubigins, theaflavins and theabrownins. Previous studies have reported that theaflavins have antioxidant effects both in vitro and in vivo and have high hydroxyl and DPPH free radical scavenging activities; on top of this, theaflavins also have anti-hyperglycemia and antibacterial effects [9,10]. For these positive health effects, black tea has been widely used in beverages and dairy products. Studies have reported that adding black tea to yogurt not only increases the nutritional composition of yogurt, but also improves the taste of yogurt without inhibiting the beneficial bacteria [11]. Cardoso et al. [12] found that kombucha produced from the fermentation of black tea extract contains rich phenolic compounds and has high antioxidant activity. In addition, Xue et al. [13] found that black tea plays an important role in the processing of egg protein gels, as it could improve the gel strength of marinated egg gel. Compared with marinated egg processed with green tea, dark tea and oolong tea, the black-tea-marinated egg had the best gel strength [14]. However, there are few reports focused on the effect of black tea powder on the antioxidant activity and gel characteristics of silver carp fish balls.

Therefore, the effects of black tea powder on the antioxidant activity and gel characteristics of silver carp fish balls were explored after freezing storage for 7 days. With the addition of 0.0%, 0.1%, 0.2% and 0.3% black tea powder, antioxidant activities including reducing capacity, DPPH, ABTS and ·OH radical scavenging were analyzed. We conducted Fourier transform infrared (FT-IR) spectroscopy analysis and environmental scanning electron microscope observation on the gel strength, textural properties, and gel structure of the fish balls. Moreover, sensory evaluation analysis was also conducted to comprehensively evaluate the quality of the fish balls. This study aims to provide theoretical basis for the application of black tea powder in the production of silver carp surimi and its products.

## 2. Results and Discussion

### 2.1. Effect of Black Tea Powder on Antioxidant Activity of Fish Balls

**Figure 1:**
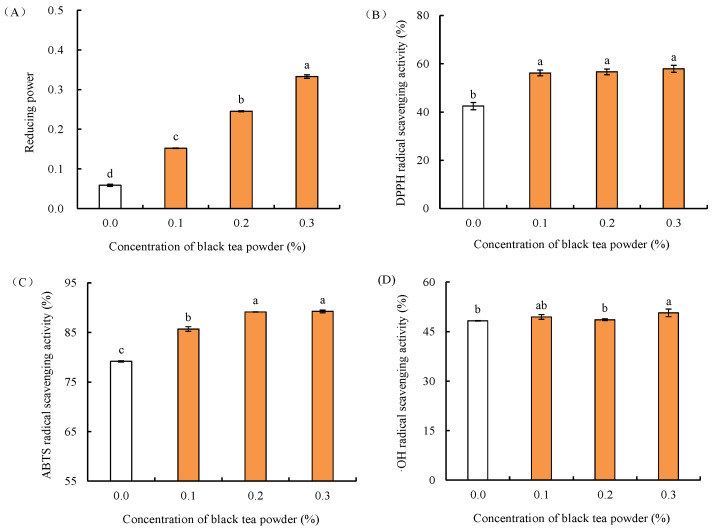
Effects of black tea powder on antioxidant activity of fish balls: (**A**) reducing power; (**B**) DPPH radical scavenging activity; (**C**) ABTS radical scavenging activity; (**D**)·OH radical scavenging activity. Data are expressed as mean ± standard deviation, *n* = 3. Different superscripts indicate significant differences (*p* < 0.05).

Surimi products are prone to accumulating oxygen free radicals during processing and freezing storage, triggering the oxidation of proteins and lipids, thus decreasing the quality of surimi products [2,3]. Generally, when the accumulation of oxygen radicals in fish balls is reduced, the oxidation of proteins in surimi products can be reduced or prevented. In addition, the theaflavins of black tea have high hydroxyl and DPPH free radical scavenging activities [9]. Reducing power, DPPH, ABTS and OH radical scavenging activity are commonly used for the determination of antioxidant activities; such methods based on radical scavenging assays are simple, rapid, helpful and inexpensive in determining the antioxidant mechanisms of a compound [15]. Therefore, the effect of black tea powder on antioxidant activities including reducing power, DPPH, ABTS and OH radical scavenging activity of surimi products during freezing was investigated. Figure 1 shows the effect of black tea powder on the antioxidant activity of fish balls. Reducing power is an indicator of antioxidant activity, which measures the ability of substance to disrupt free radical chains by supplying hydrogen [16]. As shown in Figure 1A, we found that the reducing power of fish balls with black tea powder was significantly higher than that of fish balls without black tea powder (blank group, *p* < 0.05). The reducing power of fish balls increased when the concentration of black tea powder increased. When the added concentration of black tea powder was up to 0.3%, the reducing power of fish balls was the highest (0.33) among these samples, 5.5 times the blank group (0.06). DPPH is a stable nitrogen-centered free radical widely used to detect the radical scavenging activity of compounds [17]. As shown in Figure 1B, compared with the blank group, the addition of black tea powder significantly increased the DPPH radical scavenging rate of fish balls from about 42.47% to 57.93% (*p* < 0.05). However, it was found that there was no significant difference in the DPPH radical scavenging rate of fish balls with black tea powder at the different concentrations used (*p* ≥ 0.05). The ABTS radical scavenging assay is often used to measure the antioxidant capacity of foods, where the autoxidation pathway is blocked by converting ABTS radicals to their relatively stable products, and it evaluates the antioxidant capacity of antioxidants based on blue/green ABTS^+^ solution brightening due to antioxidant reduction [18,19,20]. As shown in Figure 1C, compared with the blank group, the addition of black tea powder significantly improved the ABTS radical scavenging rate of fish balls (*p* < 0.05). As the concentration of black tea powder increased, the ABTS radical scavenging rate of the fish balls increased. This value was up to 89.24% when the added concentration of black tea powder was at 0.3%, but there was no significant difference in the ABTS radical scavenging rates of the fish balls between the added black tea powder concentrations of 0.3% and 0.2% (*p* ≥ 0.05). The hydroxyl radical (OH) is a very active reactive oxygen species (ROS) that can cause damage to the body by destroying biological macromolecules (such as DNA, lipids and proteins) [21,22]. Hydroxyl radical scavenging activity is often used to evaluate the radical scavenging ability of substances. The higher the scavenging rate is, the stronger the anti-hydroxyl radical scavenging ability is [22]. The effect of black tea powder on ·OH radical scavenging activity of fish balls is displayed in Figure 1D. When the concentrations of black tea powder were 0.1% and 0.2%, the hydroxyl radical scavenging rate of the fish balls was similar to that of the blank group (*p* ≥ 0.05). Only when the content of black tea powder was up to 0.3% did the hydroxyl radical scavenging rate of fish balls increase to 50.64%, which was much higher than that of the blank group (*p* < 0.05). These data suggest that the addition of black tea powder could enhance the antioxidant capacity of fish balls through greatly increasing reducing power, ABTS and DPPH radical scavenging abilities, especially when the concentration of black tea powder is over 0.3%.

Excessive production of free radicals, e.g., hydroxyl radicals can easily cause oxidative damage in the human body, which may lead to many degenerative diseases such as diabetes, atherosclerosis and cancer [23]. These radicals are the inevitable result of aerobic respiration; thus, food supplements containing antioxidants are of interest for health-promoting benefits by reducing oxidative damage [5,8,23]. Phenolic compounds with multiple hydroxyl groups are considered the most important bioactive compounds to resist radicals [20,24] because the interactions between phenol hydroxyl groups as hydrogen donors and radicals can enhance antioxidant ability (such as reducing power and DPPH radical scavenging activity) [24]. Previous studies reported that green tea powder can improve ferric reducing antioxidant power, DPPH oxidation inhibiting ability and ABTS radical scavenging activity of set-type yogurt mainly through the effect of green tea phenolic compounds [8,23]. Thus, in our study, the better antioxidant activity of fish balls after the addition of black tea powder might be caused by the hydroxyl groups of phenolic compounds in black tea powder.

### 2.2. Effects of Black Tea Powder on Gel Properties of Fish Balls

**Figure 2:**
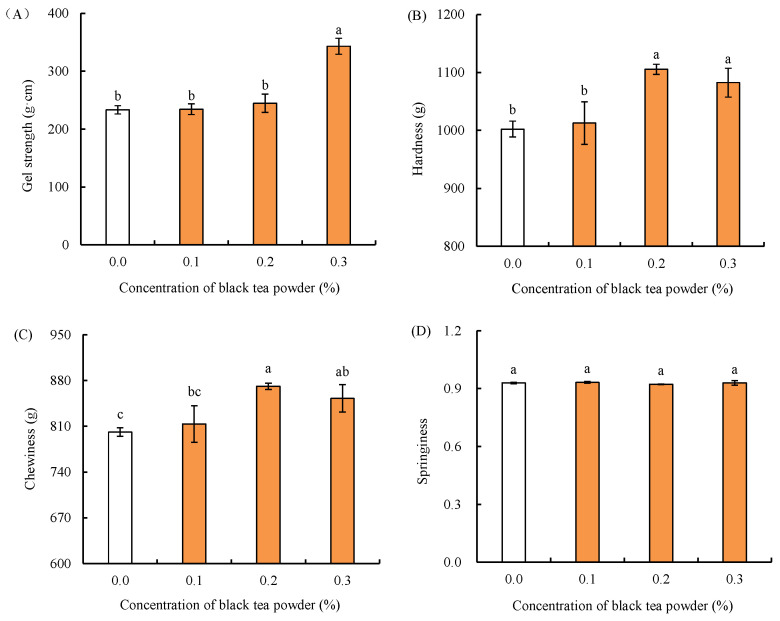
Effects of black tea powder on gel strength (**A**) and textural properties (**B**–**D**) of fish balls. Data are expressed as mean ± standard deviation, *n* = 3. Different letters indicate significant differences (*p* < 0.05).

The effect of black tea powder on gel properties including gel strength and textural properties of fish balls is shown in Figure 2. Gel strength is an important index to evaluate the gel quality of surimi products [4]. As shown in Figure 2, when the addition concentration of black tea powder was 0.1% or 0.2%, there was no significant difference in gel strength between fish balls with black tea powder and the blank group (*p* ≥ 0.05). When the addition concentration of black tea powder was 0.3%, the gel strength of the black tea powder fish balls was significantly higher than that of blank group (*p* < 0.05). Similarly, compared to the blank group, black tea powder had no effect on the hardness and chewiness of fish balls when its concentration was 0.1%, while it significantly increased those values in fish balls when the used concentration was ≥0.2% [*p* < 0.05, Figure 2B,C]. However, the addition of black tea powder had no effect on the springiness of fish balls [*p* ≥ 0.05, Figure 2D]. A previous study also reported that high concentrations of oxidant could affect the hardness of myofibrillar protein gel but had no significant effect on its springiness [25]. In summary, the addition of black tea powder, particularly at 0.3%, could promote the gel formation of fish balls with higher gel strength, hardness and chewiness.

The promotional effect of black tea powder on the gel formation of fish balls might be a result of the increased solid content in fish balls as the amounts of black tea powder increased, promoting the interaction and aggregation of surimi proteins, thus causing increased gel strength, hardness and chewiness in the fish balls [4,5]. In addition, the gel properties of fish balls may also be related to their antioxidant activity. As reported previously, antioxidants such as green tea extract with abundant phenolics not only enhance the antioxidant activity of surimi product but also improve its gel properties by inhibiting lipid and protein oxidation [5]. Cheng et al. [2] has reported that mulberry polyphenols can inhibit protein oxidation in dried minced pork slices. On the contrary, oxidation could reduce the hardness of minced beef gel [26]. As expected, the results in our study also show a potential positive correlation between the antioxidant capacity (Figure 1) and gel properties (Figure 2) of fish balls with black tea powder. Based on the above discussion, it is implied that the addition of black tea powder may potentially inhibit protein and lipid oxidation in fish balls, thus improving the gel properties of fish balls. In addition, the phenolic compounds are able to promote protein aggregation through ε-lysine crosslinking, resulting in the better gel properties of aquatic products [27,28]. Therefore, the phenolic compounds of black tea powder might be also attributed to its promotion of the gel formation effect of fish balls. In conclusion, the increased solid content, enhanced antioxidant capacity, and phenolic compounds of black tea powder are much related to the higher gel strength, hardness and chewiness of fish balls, particularly with addition of black tea powder at 0.3%.

### 2.3. Effects of Black Tea Powder on Color Properties of Fish Balls

Previous studies reported that the color, concentration and type of additive can affect the whiteness of surimi gel [29,30]. Zheng et al. [4] found that the whiteness of surimi products decreases with the increase in crude fucoidan. Zhou et al. [31] also reported that egg whites modified by tea polyphenols reduced the gel whiteness of surimi. Thus, the brown color of the black tea powder was speculated to cause a decrease in the whiteness of fish balls in our study. Accordantly, Table 1 shows that addition of black tea powder could significantly reduce brightness (L*) value and whiteness of fish balls, while greatly increase red (a*) and yellow (b*) values of fish balls when compared with the blank group (*p* < 0.05). The whiteness of the fish balls decreased as the concentration of black tea powder increased (*p* < 0.05). When the addition concentration of black tea powder was 0.3%, the whiteness of fish balls was the lowest, that is, 29.64% lower than that of the blank group. Overall, black tea powder with brown color reduced the whiteness of the fish balls in a dose-dependent manner. Since consumers may prefer bright products, it is necessary to control the right amount of black tea powder or improve the color properties of black tea powder to maintain the overall quality of fish balls in the future.

### 2.4. Effects of Black Tea Powder on FT-IR of Fish Balls

**Figure 3:**
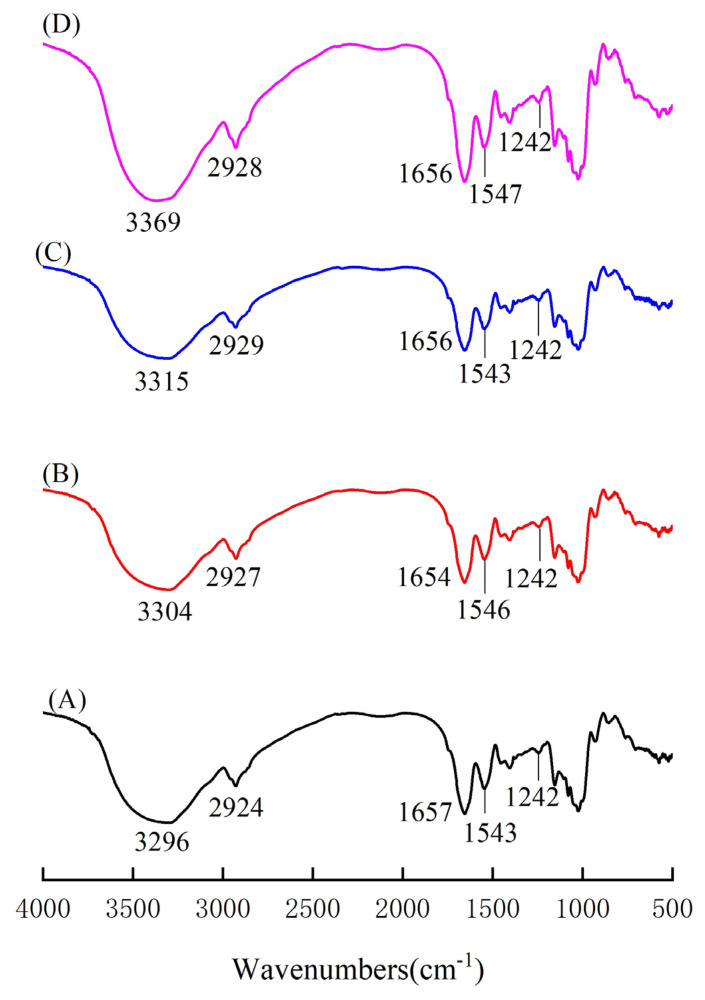
Effects of black tea powder on FT-IR of fish balls. (A) Fish ball without black tea powder; (B) fish ball with 0.1% black tea powder; (C) fish ball with 0.2% black tea powder; (D) fish ball with 0.3% black tea powder.

Figure 3 shows the effects of black tea powder on the FT-IR spectra of fish ball protein. Characteristic peaks of amide A (3296 cm^−1^) and amide B (2924 cm^−1^) have been found in fish balls [32]. From the addition of black tea powder, the characteristic peak of the amide A region gradually shifted to higher wavenumbers as the concentration of black tea powder increased, but there was no significant impact on the amide B band. This indicates that the addition of black tea powder can enhance the stretching vibration of N-H [33,34], as the addition of black tea powder could affect the interaction of -NH_2_ groups between peptide chains, leading to changes in some functional groups [35]. Meanwhile, the wavenumbers of the amide I, amide II and amide III peaks were little affected by black tea powder. However, the addition of 0.3% black tea powder increased the intensity of the amide I and amide II bands for fish balls. This might be due to the fact that phenolic compounds in black tea powder could enhance the extension of aromatic rings in amide Ⅰ and amide Ⅱ bands [36]. Similarly, when fish oil and green tea extracts were added to surimi, the strength of the amide I and amide II bands in the surimi gel protein increased with the presence of oil and phenolic compounds [5].

### 2.5. Effect of Black Tea Powder on Microstructures of Fish Balls

**Figure 4:**
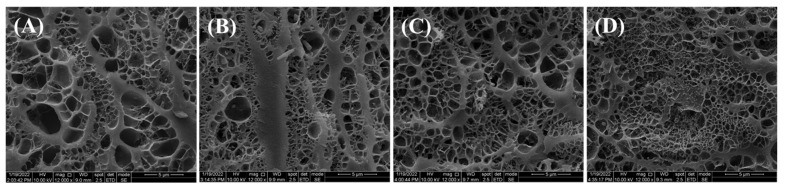
Environmental scanning electron micrograph of fish ball gels. (**A**) Fish ball without black tea powder; (**B**) fish ball with 0.1% black tea powder; (**C**) fish ball with 0.2% black tea powder; (**D**) fish ball with 0.3% black tea powder.

Microstructure can reflect the gel characteristics of fish balls such as organization, texture and function [37]. The effect of black tea powder on the microstructure of fish ball gels are shown in Figure 4. Fish balls display a porous gel structure. Compared with the blank group (Figure 4A), the pore size of fish ball gel was reduced by the addition of black tea powder, exhibiting a more compact structure as protein aggregation increased. When black tea powder was added at the level of 0.3%, the gel structure of fish balls (Figure 4D) had more uniform pore size, with a more compact and regularly ordered structure than that of fish balls with black tea powder added at 0.1% (Figure 4B) or at 0.2% (Figure 4C). Generally, dense and regularly ordered gel structure corresponds with high gel strength [32,38]. Our result of gel structure (Figure 4) was also well consistent with the above findings that gel strength, hardness, chewiness of fish balls increased from the addition of 0.3% black tea powder (Figure 2).

### 2.6. Effect of Black Tea Powder on Sensory Evaluation of Fish Balls

Table 2 shows the effect of black tea powder on sensory properties including color, flavor, tissue, springiness, taste and fondness for fish balls. The color of fish balls was significantly darkened by the color of black tea powder (*p* < 0.05). This result is well consistent with the above data (Table 1) determined via chromatic meter, suggesting that the significant decrease in whiteness determined using the chromometer was also observed by humans, which may influence consumer acceptance of the fish ball product. The springiness of fish balls did not change significantly after the addition of black tea powder (*p* ≥ 0.05), this also being in accordance with the textural properties (Figure 2D) of fish balls. However, only when the addition concentration of black tea powder rose to 0.3% did the flavor, taste and fondness for fish balls increase compared to the blank group (*p* < 0.05). In addition, the tissue state of fish balls with black tea powder was not significantly different from that of blank group (*p* ≥ 0.05). This finding was not consistent with the ESEM image results, probably because the pore size of the fish balls is too small to judge the differences with the naked eye. This indicates that the addition of black tea powder at 0.3% significantly reduced the whiteness of the fish balls but improved the fishy taste of the fish balls, making fish balls with tea flavor a way to improve consumer satisfaction.

## 3. Conclusions

In this study, the effect of black tea powder on fish ball antioxidant activity was explored using radical scavenging assays, and gel characteristics including gel strength, textural properties, whiteness and sensory evaluation were also investigated. The results show that black tea powder could significantly increase the antioxidant activity, gel strength, hardness and chewiness of fish balls (*p* < 0.05), and the microstructure of fish ball gel became more compact with denser, improved connectivity. In particular, the strongest antioxidant activity of fish balls was found at 0.3% black tea powder added, where the reducing power, DPPH, ABTS and ·OH free radical scavenging rate were about 0.33, 57.93%, 89.24% and 50.64%, respectively. This may be related to the rich phenolic compounds in black tea powder. In addition, black tea powder reduced the whiteness of fish balls and had no significant effect on the springiness of fish balls. Moreover, the mechanism of black tea powder influencing the antioxidant activity and gel characteristics of fish balls still needs further study. In conclusion, the results of this study indicate that black tea powder improved the antioxidant activity and gel characteristics of fish balls during frozen storage, providing basic guidance for the application of black tea powder in the production of surimi products.

## 4. Material and Methods

### 4.1. Materials

Frozen silver carp surimi was purchased from Fujian Anjing Foods Co., Ltd. (Xiamen, China), and kept at −20 °C until used. Black tea powder (caffeine removed) was purchased from Damin Foodstuff (Zhangzhou) Co., Ltd. (Zhangzhou, China). Tapioca starch, phosphate, salt and glutamine transaminase (TG enzyme) were commercially available.

1,1-diphenyl-2-picrylhydrazyl (DPPH) was purchased from Shanghai Yuanye Biotechnology Co., Ltd. (Shanghai, China); 2,2′-azino-bis (3-ethylbenzothiazoline-6-sulfonic acid) (ABTS) was purchased from Beijing Solarbio Technology Co., Ltd. (Beijing, China); sodium dihydrogen phosphate, dibasic sodium phosphate, trichloroacetic acid, hydrogen peroxide, ferrous sulfate and potassium persulfate were of analytical grade and purchased from Xilong Chemical Co., Ltd. (Guangzhou, China). Absolute ethanol was purchased from Sinopharm Group Chemical Reagent Co., Ltd. (Shanghai, China); ferric chloride, potassium ferricyanide, and 3,5-Dinitrosalicylic acid were purchased from Shanghai Macklin Biochemical Technology Co., Ltd. (Shanghai, China).

### 4.2. Preparation of Fish Balls

Frozen surimi was thawed at 4 °C for 12 h before cutting into small cubes. Thawed surimi with a weight of 350 g was added into a beater (Fujian Anjing Foods Co., Ltd., Xiamen, China) to chop for 2 min, and then 10 g salt was added and mixed with the surimi for 7 min. Subsequently, 50 g tapioca starch, 1 g phosphate compound (sodium tripolyphosphate: sodium pyrophosphate: sodium hexametaphosphate = 1:1:1), and 0.0% (blank group), 0.1%, 0.2% or 0.3% black tea powder (*w*/*w* raw materials) were added. Next, 0.5 g TG enzyme and 90 g ice water were added and mixed with the above surimi for 6 min. In the pre-experiment, the sensory evaluation of fish balls with black tea powder was carried out. It was found that the umami taste of silver carp was weakened when 0.3% black tea powder was added, so its maximum addition amount was set to 0.3%. The evenly mixed slurry was made into fish balls, pre-heated at 45 °C for 30 min, then heated at 90 °C for 10 min using a water bath (HH. S 21-4, Shanghai Boxun Medical Bio-Instrument Co., Ltd., Shanghai, China) and cooled in ice water for 20 min. The cooled fish balls were packaged and stored at −18 °C for 7 days.

### 4.3. Antioxidant Activity Analysis

The fish balls were blended with distilled water at 1:20 *w*/*v* and homogenized for 5 min. The samples were centrifuged at 2021× *g* for 5 min using a refrigerated centrifuge (Sigma-18K, Sigma Laborzentrifugen GmbH, Braunschweig, Germany) to obtain supernatant, and then filtered through vacuum suction. The supernatant was freeze-dried in a vacuum freeze dryer (Free Zone 6 plus, American Labconco Co., Ltd., Downtown Kansas City, MO, USA). After freeze-drying, 200 mg of each sample was redissolved in 10 mL distilled water, and then the antioxidant activity experiments were carried out [4].

#### 4.3.1. Reducing Capacity

Reducing capacity was determined according to a method reported previously [21] with some modifications. Using the potassium ferricyanide reduction method, each 0.5 mL homogenate of fish balls was mixed with 0.5 mL phosphate buffer (0.2 mol/L, pH = 6.6) and 0.5 mL K_3_Fe(CN)_6_ solution (1%, *w*/*v*), reacting at 50 °C for 20 min in a water bath. After cooling to room temperature, 0.5 mL trichloroacetic acid solution was added (10%, *w*/*v*), and then the solution was centrifuged at 13,201× *g* for 5 min. After centrifugation, 0.5 mL of supernatant was mixed with 0.5 mL distilled water and 100 μL FeCl_3_ solution (0.1%, *w*/*v*) to react at 50 °C for 10 min; then, absorbance values were measured at 700 nm. The following Equation (1) was used to calculate reducing capacity:

(1) $$\mathrm{Reducing}\;\mathrm{capacity}=A_{1}-A_{0}$$

where A_1_ is the absorbance of the sample and A_0_ is the absorbance of the blank (using distilled water instead of homogenates).

#### 4.3.2. DPPH Radical Scavenging Activity

DPPH radical scavenging activity was measured according to a previously described method [39] with some modifications. Each 0.8 mL homogenate of fish balls was mixed with 0.8 mL DPPH ethanol solution (0.1 mmol/L), reacting at room temperature for 30 min, and then centrifuged at 13,201× *g* for 5 min. Finally, absorbance values were measured at 517 nm. DPPH radical scavenging activity was calculated by using Equation (2):

(2) $$DPPH\;radical\;scavenging\;rate\;\left(\%\right)=\left(1-\frac{A_{1}}{A_{0}}\right)\times 100\%$$

where A_1_ is the absorbance of the sample and A_0_ is the absorbance of the blank (using distilled water instead of homogenates).

#### 4.3.3. ABTS Radical Scavenging Activity

The ABTS radical scavenging activity of fish balls was performed based on the method described previously [40] with some modifications. ABTS solution (7 mmol/L), K_2_S_2_O_8_ solution (2.4 mmol/L) and phosphate buffer (0.2 mol/L, pH = 7.0) were prepared. ABTS solution was mixed with K_2_S_2_O_8_ solution in equal proportions, and then the mixture was placed in the dark at room temperature for 12–16 h to prepare ABTS^+^ stock solution. The ABTS^+^ stock solution was diluted with phosphate buffer when in use until its absorbance value was 0.7 ± 0.02 at a wavelength of 734 nm. Each 0.1 mL homogenate of fish balls was mixed with 1 mL ABTS^+^ stock solution and heated at 37 °C for 1 h via water bath. The homogenate was centrifuged at 13,201× *g* for 5 min, after which the absorbance was determined at a wavelength of 734 nm. The ABTS radical scavenging rate was calculated by using Equation (3):

(3) $$ABTS\;radical\;scavenging\;rate\;\left(\%\right)=\left(1-\frac{A_{1}}{A_{0}}\right)\times 100\%$$

where A_1_ is the absorbance of the sample and A_0_ is the absorbance of the blank (using distilled water instead of homogenates).

#### 4.3.4. OH Radical Scavenging Activity

Referring to the method of Chen and Huang [22], 0.1 mL homogenate of fish balls, 0.1 mL salicylic acid ethanol solution (9 mmol/L), 0.1 mL FeSO_4_ solution (9 mmol/L), 0.6 mL deionized water and 0.1 mL H_2_O_2_ (8.8 mol/L) were blended in order. The mixture reacted at 37 °C for 10 min in a water bath, followed by centrifuging at 13,201× *g* for 5 min. Finally, their absorbances were determined at a wavelength of 510 nm. The OH radical scavenging activity was calculated by using Equation (4):

(4) $$\cdot OH\;radical\;scavenging\;rate\;\left(\%\right)=\left(1-\frac{A_{1}}{A_{0}}\right)\times 100\%$$

where A_1_ is the absorbance of the sample; A_0_ is the absorbance of the blank (using distilled water instead of homogenates).

### 4.4. Determination of Gel Strength

We cut the fish balls into approximately 2 × 2 × 2 cm and used a texture analyzer (TA.XT. plus, Stable Micro System, Surrey, UK) with a P/5s probe to analyze the gel strength of the fish balls [41]. The measuring parameters were as follows: the mode was set as compression, the displacement was 10 mm, and the trigger force was 5 g; the speed before and after the test were both 2 mm/s, the test speed was 1 mm/s. Gel strength was calculated by using Equation (5):

(5) Gel strength(g⋅cm)=breaking force×breaking strain

### 4.5. Determination of Texture Profile Analysis (TPA)

Following the method of Li [42,43], the fish balls were cut into approximately 2 × 2 × 2 cm and a texture analyzer (TA.XT. plus, Stable Micro Systems, Surrey, UK) was used to determine texture parameters including hardness, elasticity, and chewiness of the fish balls. The probe of P 36R was used, and the testing was performed under TPA mode. The test speed was 1 mm/s, the deformation was 30%, the speeds before and after the test were both 2 mm/s, and the trigger force was 5 g. Hardness reflects the maximum force required to deform the surimi gels; chewiness indicates the required energy to chew the surimi gel into a state that can be swallowed, equivalent to the product of hardness, cohesiveness and springiness; springiness indicates the ability of the surimi gel to deform and return to its original shape and size after the removal of external forces.

### 4.6. Determination of Color Properties

Color measurement of fish balls was evaluated using a chromatic meter (WSC-S, Shanghai INESA Optical Instrument Co., Ltd., Shanghai, China) following Li [42]. L* (brightness), a* (+*a*, red; −*a*, green) and b* (+*b*, yellow; −*b*, blue) were determined. Whiteness was calculated by using Equation (6):

(6) $$Whiteness=100-{\left[{\left(100-L*\right)}^{2}+{a*}^{2}+{b*}^{2}\right]}^{\frac{1}{2}}$$

### 4.7. Fourier Transform Infrared (FT-IR) Spectroscopy Analysis

According to a previous method of Guan [34], the freeze-dried fish balls were crushed into fine powder, mixed with potassium bromide powder at 1:100 (*w*/*w*), ground evenly and pressed. An infrared spectrometer (Nicolet iS50, Thermo Fisher Scientific, Waltham, MA, USA) was used to scan samples within the range of 4000–400 cm^−1^.

### 4.8. Gel Microstructure Analysis

The fish balls were cut into small pieces and coated with conductive carbon glue. The fish balls were frozen in liquid nitrogen for 30 s, then transferred to the sample preparation chamber under vacuum using a cryogenic freeze preparation transport system. The samples were sublimated, gilded, and observed by using an environmental scanning electron microscope (Quanta 450, FEI Co., Ltd., Portland, OR, USA) [4]. The objective used for all experiments was at 1.2 × 10^4^ magnification.

### 4.9. Sensory Evaluation

Sensory evaluation of fish balls was based on a previous report [44] with some modifications. Fish balls were sliced and equilibrated at room temperature for 30 min. The evaluation team consisted of 10 sensory evaluation trainees (5 women and 5 men) who had a professional food background and had received professional sensory training. According to the sensory evaluation standards in Table 3, the color, odor, tissue, springiness, taste and fondness towards the fish balls were evaluated.

### 4.10. Statistical Analysis

The data were calculated and plotted using Microsoft Excel software, using SPSS Statistics 26 software for one-way analysis of variance (ANOVA), and we used Duncan multiple comparisons to compare the significance of each datum, *p* < 0.05.

## Figures and Tables

**Table 1:** Effect of black tea powder on color properties of fish balls.

Concentration/%	L*	a*	b*	Whiteness
0.0	61.00 ± 0.39 ^a^	0.58 ± 0.28 ^d^	4.67 ± 0.16 ^d^	60.72 ± 0.41 ^a^
0.1	53.30 ± 0.67 ^b^	6.78 ± 0.21 ^c^	7.77 ± 0.16 ^c^	52.17 ± 0.66 ^b^
0.2	49.00 ± 0.41 ^c^	10.88 ± 0.55 ^b^	9.86 ± 0.35 ^b^	46.93 ± 0.46 ^c^
0.3	45.72 ± 0.54 ^d^	14.83 ± 0.45 ^a^	10.68 ± 0.51 ^a^	42.72 ± 0.46 ^d^

Note: Data are expressed as mean ± standard deviation. Different letters indicate significant differences (*p* < 0.05).

**Table 2:** Effect of black tea powder on the sensory evaluation of fish balls.

Project	0.0%	0.1%	0.2%	0.3%
Color	9.25 ± 0.43 ^a^	7.25 ± 0.43 ^b^	5.88 ± 0.78 ^c^	4.50 ± 1.32 ^d^
Odor	5.50 ± 1.12 ^b^	7.06 ± 1.24 ^ab^	6.63 ± 1.65 ^ab^	7.38 ± 1.49 ^a^
Tissue	6.75 ± 1.48 ^a^	7.13 ± 1.17 ^a^	6.63 ± 1.11 ^a^	5.88 ± 1.54 ^a^
Springiness	8.38 ± 0.99 ^a^	8.63 ± 0.86 ^a^	7.88 ± 1.27 ^a^	8.25 ± 0.83 ^a^
Taste	6.25 ± 1.09 ^b^	7.50 ± 1.32 ^ab^	7.19 ± 1.46 ^ab^	7.88 ± 1.27 ^a^
Fondness	6.00 ± 1.00 ^b^	7.13 ± 1.54 ^ab^	7.25 ± 1.48 ^ab^	7.88 ± 0.60 ^a^

Note: Data are expressed as mean ± standard deviation. Different letters indicate significant differences (*p* < 0.05).

**Table 3:** Criteria for sensory evaluation.

Project	Standard Description	Score
Color	Reddish brown, with no gloss	0~4
	Yellowish brown, the gloss is slightly darker	5~6
	Light brown, relatively shiny	7~8
	Gray, shiny	9~10
Odor	Strongly fishy	0~4
	Obviously fishy	5~6
	Slightly fishy	7~8
	Not fishy	9~10
Tissue	Internal structure with loose and larger pores	0~4
	Loose internal structure with many small pores, and uniform	5~6
	Compact internal structure, with tiny and uniform small pores	7~8
	Compact internal structure, without small pores	9~10
Springiness	Cracks when pressed	0~4
	Does not crack after pressing and cannot be completely restored to its original shape after press removal	5~6
	Does not crack after pressing and cannot rapidly recover after press removal	7~8
	Does not crack after pressing and can rapidly recover after press removal	9~10
Taste	No fish taste and rough chew	0~4
	Less fish taste and slightly rough chew	5~6
	With fish taste and tea taste, slightly delicate and smooth	7~8
	With fish taste and tea taste, delicate and smooth	9~10
Fondness	Don’t like	0~4
	General	5~6
	Prefer	7~8
	Like very much	9~10

## Data Availability

The data presented in this study are available on request from the corresponding author.
